# Electrical and optical properties of binary CN_*x*_ nanocone arrays synthesized by plasma-assisted reaction deposition

**DOI:** 10.1186/1556-276X-9-135

**Published:** 2014-03-21

**Authors:** Xujun Liu, Leilei Guan, Xiaoniu Fu, Yu Zhao, Jiada Wu, Ning Xu

**Affiliations:** 1 Shanghai Engineering Research Center of Ultra-Precision Optical Manufacturing, Department of Optical Science and Engineering, Fudan University, Shanghai 200433, People's Republic of China

**Keywords:** CN_*x*_ nanocone arrays, Wideband absorption, Electrical conduction, Wettability to polymer absorbers, 81.07.Bc, 61.46.Np, 52.77.Dq

## Abstract

Light-absorbing and electrically conductive binary CN_*x*_ nanocone (CNNC) arrays have been fabricated using a glow discharge plasma-assisted reaction deposition method. The intact CNNCs with amorphous structure and central nickel-filled pipelines could be vertically and neatly grown on nickel-covered substrates according to the catalyst-leading mode. The morphologies and composition of the as-grown CNNC arrays can be well controlled by regulating the methane/nitrogen mixture inlet ratio, and their optical absorption and resistivity strongly depend on their morphologies and composition. Beside large specific surface area, the as-grown CNNC arrays demonstrate high wideband absorption, good conduction, and nice wettability to polymer absorbers.

## Background

Since the 1990s, there has been an upsurge in interest in the properties and potential uses of carbon-related nanostructures [1-3]. These unique nanostructures are attractive for nanotechnology applications in photovoltaic devices and photodetectors [4-8]. Many novel thin film solar cells rely on highly light-absorbing and well electrically conductive electrodes for their successful operation and good capability. For example, dye-sensitized solar cells and polymer organic hybrid solar cells exploit titanium oxide as electrodes [7,8]. But, this material is far from ideal because of poor electrical conduction and limited optical absorption [9,10]. Carbon-related nanostructures, such as carbon nanotubes and graphene, are attractive electrodes and even absorbers for photovoltaic devices and photodetectors owing to strong optical absorptivity and ultrafast charge transport mobility [6,11]. Besides, their large specific surface area could greatly increase the donor/acceptor interface, which will effectively increase the separation probability of electrons and holes. Compared with carbon nanotubes and graphene, the binary CN_*x*_ nanocones (CNNCs) will have good mechanical stability and better electrical and chemical stabilities due to the incorporation of nitrogen. So far, the experimentally synthesized carbon nitride, except our previous reports of the growth of the CNNC arrays [12], is mainly limited to amorphous or nanosphere CN_*x*_ thin films and nanobells with low nitrogen content (about 2%) [13-15].

Here, vertically aligned CNNC arrays with high wideband absorption and good electrical conduction were fabricated by an abnormal glow discharge plasma-assisted reaction deposition (GPRD) method which combines highly dense plasma with proper bias enhancement [12]. The methane/nitrogen (CH_4_/N_2_) mixture feeding gas ratio, which directly affected the contents and activities of the nitrogen-related and carbon-related precursors in the plasmas, was regulated to control the morphologies and composition of the CNNC arrays. The effects of the morphology, composition, and structure of the CNNC arrays on their optical absorption and electrical conduction were studied. The CNNC arrays with intact shape, high optical absorption, high electrical conduction, and nice wettability to polymer are pursued for potential uses as electrodes or even absorbers in photovoltaic devices and photodetectors.

## Methods

Optically absorptive and electrically conductive CNNC arrays were grown on nickel-covered silicon (100) substrates by means of the GPRD method, as described previously [12,16]. The sample preparation involves two steps. In the first step, nickel catalyst layers were deposited on silicon (100) wafers by a pulsed laser deposition method. About 100-nm thick nickel catalyst layers were deposited on the prepared substrates under a base pressure of 1 × 10^-3^ Pa for 8 min using a Nd:YAG laser to ablate a pure nickel target. The wavelength, pulse energy, and repetition of the Nd:YAG laser were 532 nm, 50 mJ, and 10 Hz, respectively. The distance between the target and substrate was about 4 cm. In the second step, the CNNC arrays were grown by the GPRD method. The plasma source generated reactive plasma just above the substrates through the abnormal glow discharge with a CH_4_/N_2_ mixture inlet under a total pressure of 750 Pa. The discharge current, voltage, and time were set to 180 mA, 350 V, and 40 min, respectively. In the CNNC growth, the CH_4_/N_2_ inlet ratios were varied from 1/80 to 1/5 in order to obtain the CNNC arrays with different morphologies and compositions. The wettability of the CNNC arrays to poly-3-hexylthiophene mixed with phenyl-C61-butyric acid methyl ester (P3HT:PCBM) layer, which is a commonly used polymer absorber in polymer organic hybrid solar cells, has also been examined by spin coating method using different rotational speeds for different polymer thicknesses.

The morphologies of the samples were characterized by field emission scanning electron microscopy (FESEM) and transmission electron microscopy (TEM). The crystallinity and composition of the individual CNNCs were characterized by selected-area electron diffraction (SAED) and energy-dispersive X-ray spectroscopy (EDXS). The optical absorption spectra were measured by an ultraviolet spectrophotometer. Longitudinal resistance of the as-grown CNNC arrays was measured by a platinum-cylindrical-tip contacting method using a Power SourceMeter (Keithley Instruments Inc., Beijing, China), and the resistivity of the as-grown CNNCs was obtained by calculating the measured resistance.

## Results and discussion

**Figure 1:**
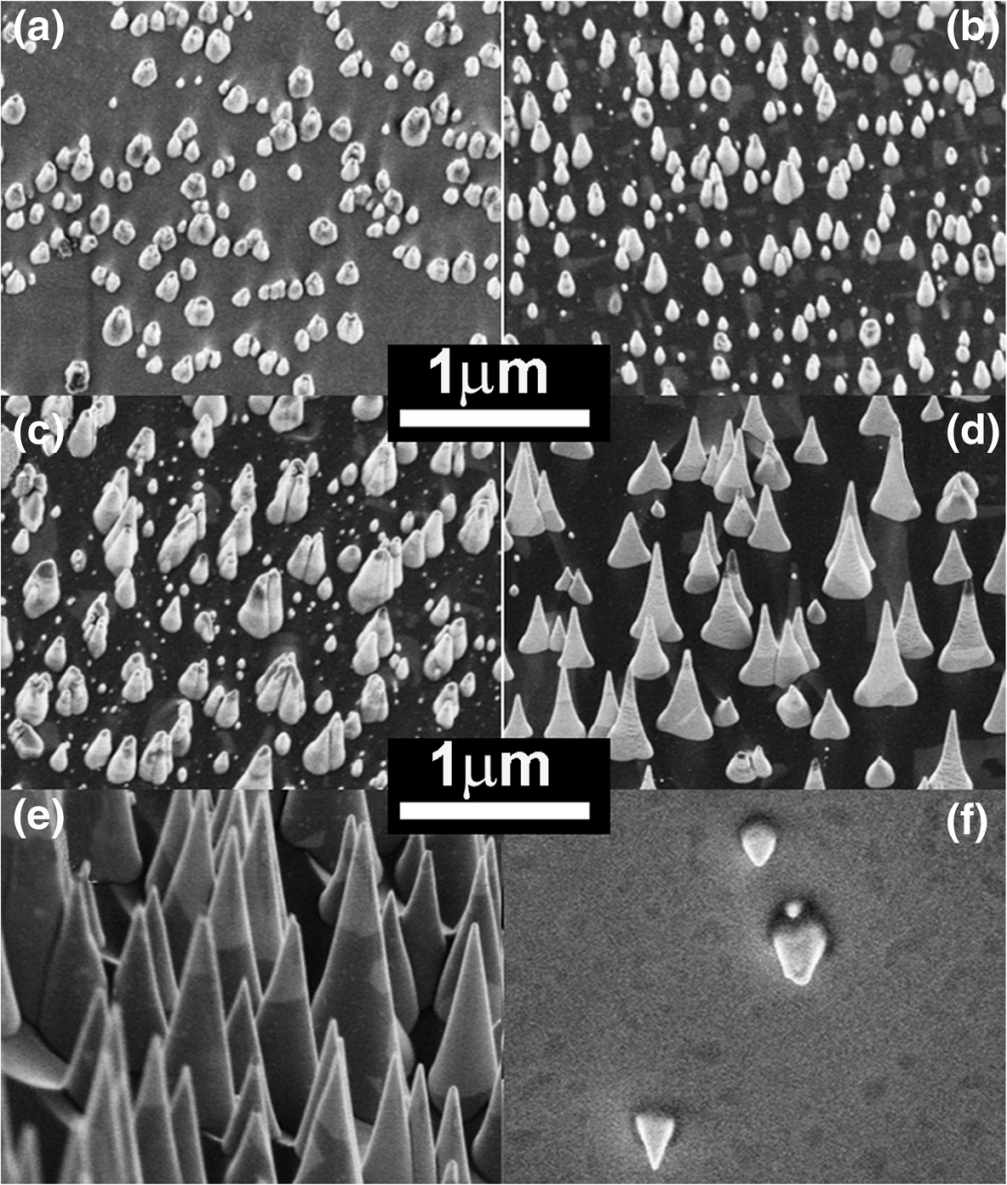
**FESEM images of the CNNC arrays grown at different CH**_**4**_**/N**_**2 **_**feeding gas ratios. (a)** 1/80, **(b)** 1/40, **(c)** 1/20, **(d)** 1/10, **(e** and **f)** 1/5. **(f)** The surface morphologies of the P3HT:PCBM-covered CNNC arrays grown at a CH_4_/N_2_ feeding gas ratio of 1/5. The samples were prepared on the nickel-covered silicon (100) wafers for 40 min, with a discharge current of 180 mA and a discharge voltage of 350 V.

The FESEM images of the CNNC arrays grown at different CH_4_/N_2_ feeding gas ratios of 1/80 to 1/10 are shown in Figure 1. It is apparent in Figure 1 that the morphologies and sizes of the as-grown CNNCs are strongly dependent on the CH_4_/N_2_ ratios. Figure 1a shows that there are almost no intact CNNCs, but many dispersive hemispherical clusters were clearly discerned when the CH_4_/N_2_ ratio is 1/80. These CNNCs are in the incomplete-growth stage. As the CH_4_/N_2_ ratio was increased, the sizes of the as-grown CNNCs were increased and their morphologies were improved (Figure 1c,d,e). It can be seen in the Figure 1e that the CNNCs grown at the CH_4_/N_2_ ratio of 1/5 have rather perfect shape, and their average bottom diameter, average height, and identical apex angle are about 400 nm, 1,000 nm, and 25°, respectively. By comparing the five images (Figure 1a,b,c,d,e), it could be found that the average height and bottom diameter of the as-grown CNNCs increase quickly, but their distribution density changes inapparently as the CH_4_ feeding gas increases. The above phenomena could be explained by that the supersaturation conditions necessary for the nucleation of the CNNCs could be more easily satisfied for a very little CH_4_ supply [17]. When the CH_4_ supply increases, the CN radicals in the plasma also increase and the N_2_^+^ or N^+^ etching effects become weaker relatively, which will lead to the increment of the growth rate of the CNNCs and their more intact conical shape (Figure 1d,e).

For novel thin film solar cells, such as polymer inorganic hybrid solar cells, the electrodes made from inorganic nanostructures not only require high optical absorption and good electrical conduction but also nice wettability to absorbers, which is almost the main bottleneck of the development of this kind of solar cells. The wettability of the CNNC arrays to P3HT:PCBM (weight ratio of 1:0.8), which is a commonly used polymer absorber in polymer organic hybrid solar cells, was examined by the spin coating method. Figure 1f gives the FESEM image of the surface morphology of the P3HT:PCBM-covered CNNC array. It could be seen in Figure 1f that the P3HT:PCBM layer have fully infiltrated the CNNC arrays, and the several higher CNNC tips protrude from the P3HT: PCBM layer, which indicates that the CNNC arrays have very nice wettability to the P3HT:PCBM absorber layers.

**Figure 2:**
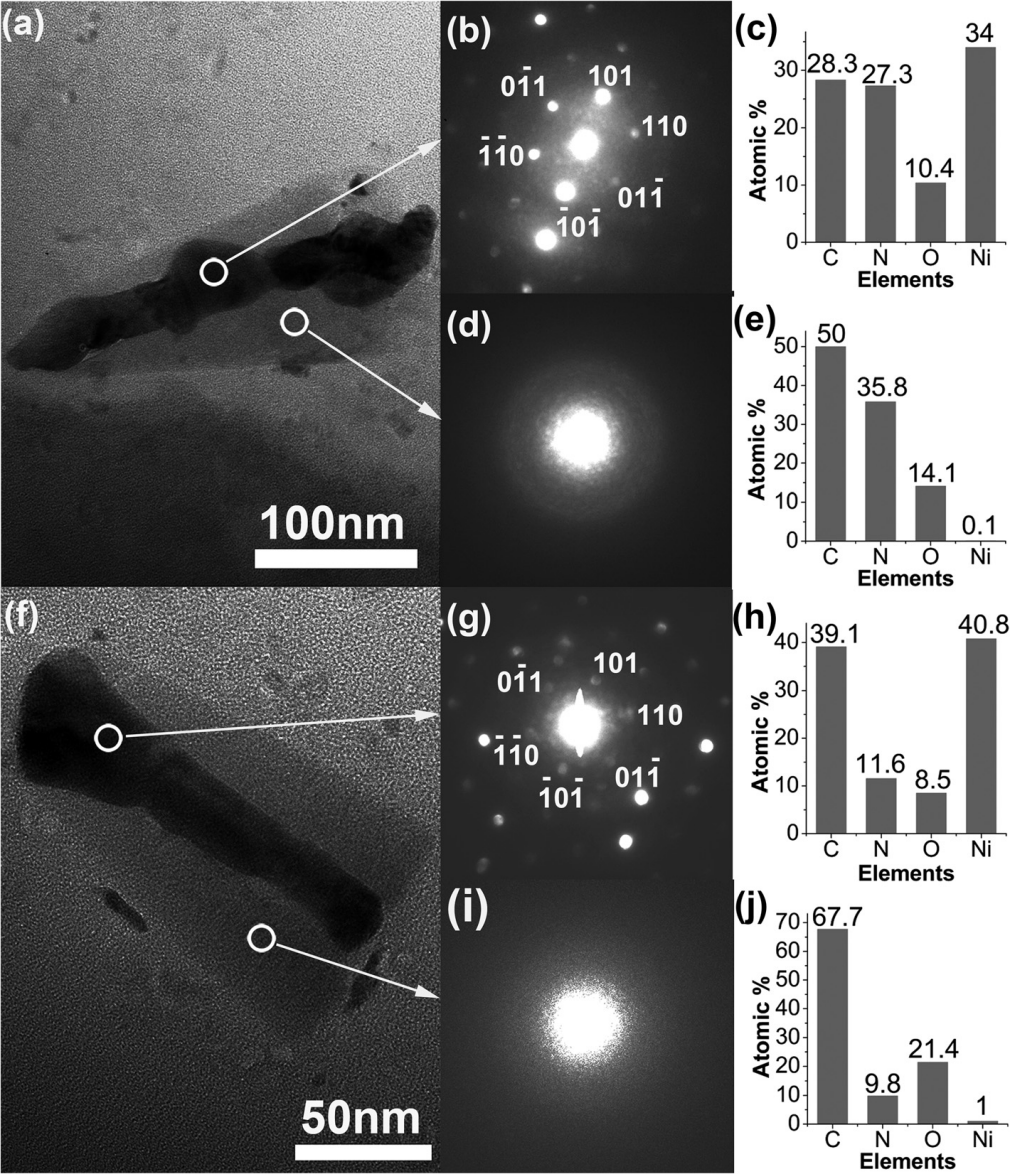
**TEM images, SAED patterns, and EDXS analytical histograms.** TEM images of the individual CNNCs prepared at the CH_4_/N_2_ feeding gas ratios of **(a)** 1/20 and **(f)** 1/5, SAED patterns taken from the black areas of the central pipes **(b** and **g)** and the lateral gray areas **(d** and **i)**, and EDXS analytical histograms **(c**, **e**, **h**, and **j)** at the locations corresponding to subgraphs **(b**, **d**, **g**, and **i)**, respectively.

In order to understand the detailed structures and composition of the CNNCs, the TEM, SAED, and EDXS fitted within the TEM were carried out. The TEM images of the two CNNCs grown at the CH_4_/N_2_ ratios of 1/20 and 1/5 are presented in Figure 2a,f. The individual CNNCs were directly scraped off from the sample surfaces and transferred onto the copper grids covered by about 10-nm carbon thin films for TEM observation. The grown CNNCs displayed good mechanical stability and strong adhesion to the substrates for the samples need to be forcibly scratched with a steel knife to obtain very few scraped-off CNNCs. Figure 2a,f shows that there are hollow pipes along the centric axes in the broken CNNCs, and they are completely filled with a kind of black substance, which have obvious contrast with the lateral areas. The SAED patterns demonstrate that the black substance in the central pipes contains crystalline nickel with a face-centered cubic structure (as shown in Figure 2b,g), and the gray substance in the lateral areas is mainly amorphous (as shown in Figure 2d,i). Some diffraction spots can be perceived in Figure 2d, but it is difficult to distinguish their crystal lattice. The analytical results of the EDXS spectra taken from the locations corresponding to Figure 2b,g also show that the atomic percentages of nickel at the central black pipes are highest in all ingredients (Figure 2c,h). Because the electron beam for X-ray analysis can easily penetrate the CNNC bodies, the partial carbon and nitrogen shown in Figure 2c,h should come from the CNNC bodies in the front and rear of the central pipes, and the nickel content in the central pipes should be more. In Figure 2e,j, it could be found that the CNNC bodies at the gray areas are mainly composed of [C] and [N], and the atomic percentages of nickel are below 0.1%. Here, the oxygen is inevitably and should mainly come from the exposure to air for days. After deducting the contribution of the 10-nm carbon thin films on the copper grids (compared with the 50-nm CNNC thickness that the X-ray pass through), the actual atomic ratios of [N]/[C] in the CNNC bodies (given in Figure 2e,j) can reach about 0.89:1 and 0.18:1, respectively. There may be crystalline C_3_N_4_ structures at the places adjacent to the central nickel-filled pipes for the actual [N]/[C] which can reach 1.2:1 and 0.4:1 at the CH_4_/N_2_ ratios of 1/20 and 1/5 (not show here), respectively, significantly higher than elsewhere. But, because the contents of the crystalline C_3_N_4_ structures near the central pipes are not enough, it is still difficult to distinguish their crystal lattice in the SAED patterns. Because the EDXS is only a semi-quantitative analysis tool, its analysis results usually have some deviation from the actual situation. From the above SAED and EDXS results, it could be certain that the main CNNC bodies are amorphous CN_*x*_, and the [N] content in them synchronously decreases as the CH_4_/N_2_ ratio increases.

Based on the characterization of morphologies, structures, and composition, the CNNC growth can be outlined as the catalyst-leading growth mode. In this mode, the nickel catalyst layer first melts and fragments into separated hemisphere-like islands under heating of the abnormal glow discharge plasma over the substrate. Then, the incipient CNNCs are formed on the nickel islands due to the deposition of precursors such as CN species, nitrogen atoms, and C_2_ species from the discharge plasma [17]. As the CN radicals and other reactive species continue to attach, the heights and lateral diameters of the CNNCs increase simultaneously. Meanwhile, the enclosed molten nickel will be sucked to the top and leave the narrow pipelines in the center of the cone bodies by the capillary effect. The catalyst nickel on the tops will lead to the growth of the CNNCs. As the CNNCs increase in height, the ion streams accelerated by a voltage of 350 eV will be focused on the tops by a locally enhanced electric field. The intense ion streams will sputter off the attached species and cut down the diameters of the tops [18]. In this way, the intact CNNC arrays with central pipelines and sharp tips eventually finish the growth. Because the precursors are mainly composed of CN species, nitrogen atoms, and C_2_ species [17], the bodies of the as-grown CNNCs are mainly amorphous CN_*x*_ other than crystalline C_3_N_4_ which needs the reaction between atomic C and N without other species involved.

**Figure 3:**
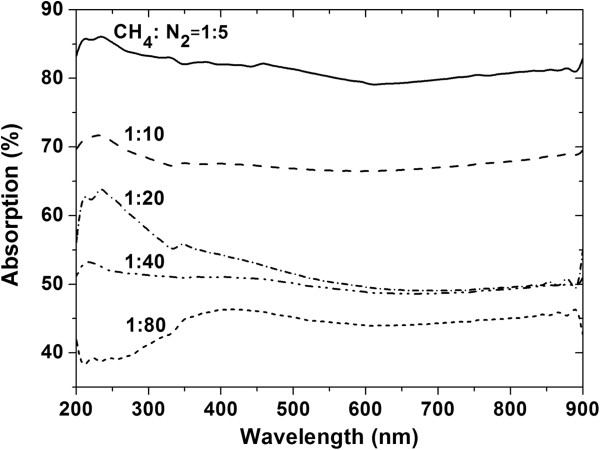
**Absorption spectra of the CNNC arrays grown at different CH**_**4**_**/N**_**2 **_**feeding gas ratios.** The CH_4_/N_2_ feeding gas ratios were 1/80, 1/40, 1/20, 1/10, and 1/5, respectively.

The optical absorption properties of the CNNC arrays are important for their application in optoelectronic devices. The optical absorption spectroscopy results of the CNNC arrays grown at CH_4_/N_2_ ratios of 1/80 to 1/5 were examined using a UV spectrophotometer in the wavelength range from 200 to 900 nm (as shown in Figure 3). It could be seen in Figure 3 that the optical absorption in the wideband of 200 to 900 nm increases as the CH_4_/N_2_ ratio increases. As the CH_4_/N_2_ ratio increased to 1/5, the absorption of the as-grown CNNC array increased to 78% to 86% in a wideband of 200 to 900 nm. By comparing the five absorption spectra, it could be found that the absorption has a larger increment rate when the CH_4_/N_2_ ratio increases from 1/20 to 1/5. This phenomenon should be mainly caused by the increase of the light refraction and repeated absorption between the CNNCs. At the CH_4_/N_2_ ratio below 1/20, the light refraction between the small and sparse CNNCs has no apparent effect on the absorption, and the absorption is mainly by base layers. Besides, there is a stronger absorption band between 200 and 400 nm for the sample prepared at the CH_4_/N_2_ ratio of 1/20, but it becomes weak when the CH_4_/N_2_ ratios are higher or lower. This absorption band may be caused by C_3_N_4_ phases (the band gaps of the α- and β-C_3_N_4_ are 3.85 and 3.25 eV, respectively) in the as-grown CNNCs [19]. The less C_3_N_4_ phases in the CNNC arrays grown at the higher CH_4_/N_2_ ratios make it weaker, while the small and sparse CNNC arrays grown at the lower CH_4_/N_2_ ratios have no significant absorption (the absorption is mainly by base layers).

**Figure 4:**
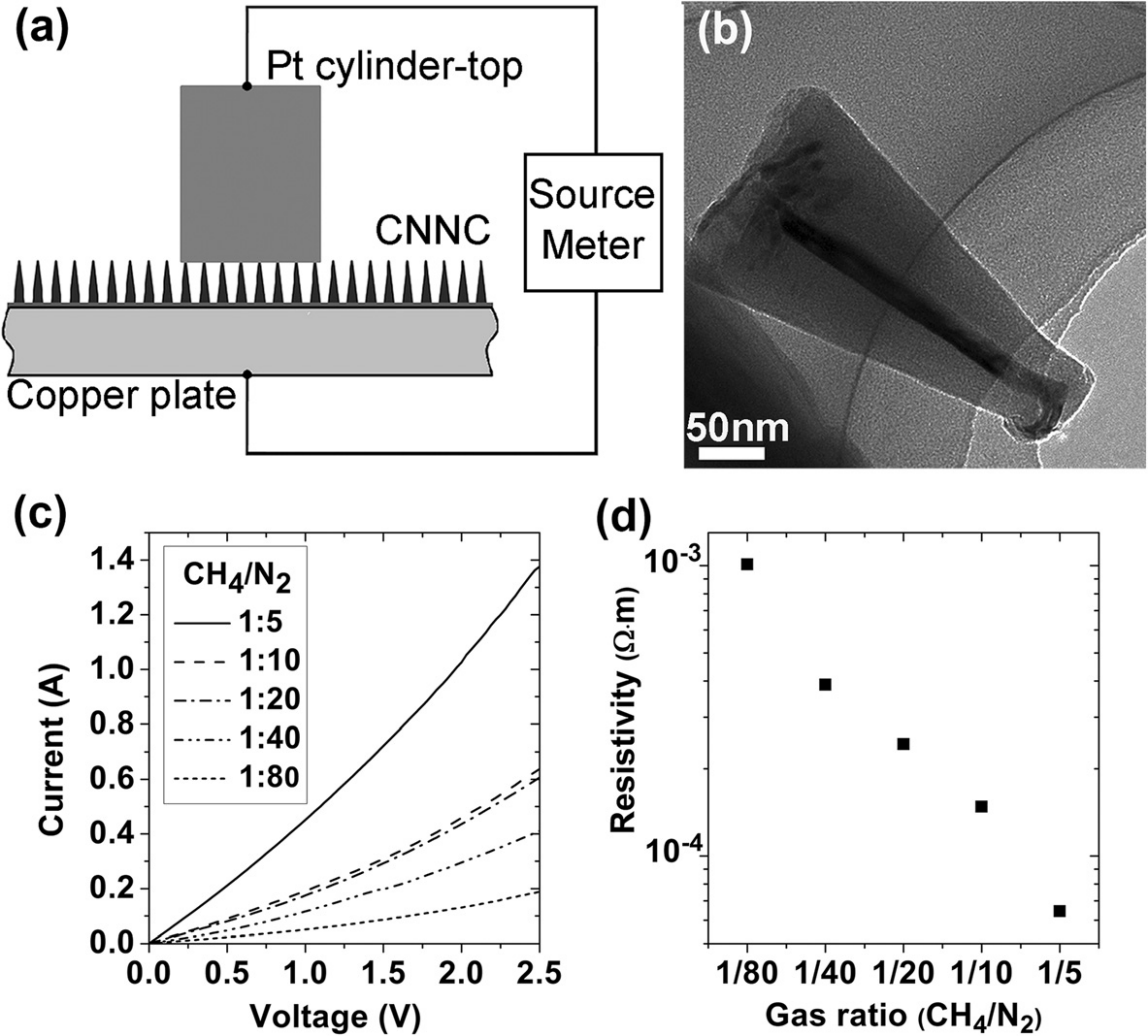
**Electrical testing diagram, TEM micrograph, *I*-*V *curves, and the corresponding resistivities. (a)** Electrical testing diagram of the CNNC arrays; **(b)** TEM micrograph of a CNNC pressed by the platinum cylindrical tip; **(c** and **d)***I*-*V* curves and the corresponding resistivities of the samples prepared at CH_4_/N_2_ feeding gas ratios of 1/80, 1/40, 1/20, 1/10, and 1/5.

For the CNNC arrays used as the electrodes of photovoltaic devices and photodetectors, their electrical properties become very important. Longitudinal resistances of the prepared CNNC arrays were measured by a platinum-cylindrical-tip contacting method. In the method, the top surface of the platinum cylindrical tip with a diameter of 1 mm directly contacted the CNNC arrays. The electrical testing diagram of the CNNC arrays is shown in Figure 4a, and the TEM micrograph of a CNNC pressed by the platinum cylindrical tip is shown in Figure 4b. The current-voltage (*I*-*V*) curves for the samples prepared at different CH_4_/N_2_ ratios of 1/80 to 1/5 are shown in Figure 4c. All *I*-*V* curves are nearly consistent with linear characteristics, and the resistance values in a circular area with a diameter of 1 mm can be obtained by fitting the corresponding slanted lines. According to the distribution density and average size of the CNNCs (estimated through the FESEM and TEM images of the as-prepared samples), the resistivities *ρ* of the as-grown CNNCs at different CH_4_/N_2_ ratios can be calculated by the following equation:

$$\rho =\frac{\mathrm{nR}}{\int_{h1}^{h2}\frac{\mathrm{dh}}{\pi {\left(h\tan\frac{\theta }{2}\right)}^{2}}},$$

where *R* is the resistance value in a circular area with a diameter of 1 mm, *n* is the number of CNNCs in the area contacted by the platinum cylindrical tip, *h*_2_ is the average height of the nanocones, *h*_1_ is the average loss height caused by the contact with the platinum cylindrical tip, and *θ* is the cone angle. According to the measured resistance (Figure 4c), the resistivity of the as-grown CNNCs can be calculated, and the results are shown in Figure 4d. In the above calculations, the impacts of the Ni-containing substances in the central pipes on the resistance are not considered. Actually, the middle sections of most central pipes (if not all) are empty due to thermal expansion and contraction, and sometimes the central pipes at the tips are also empty by TEM observations (we have not observed the whole central pipes filled by the black substances), i.e., the Ni-containing substances in the central pipes are disconnected. Besides, the resistivity of the Ni-containing substances in the central pipes is uncertain for the atomic percentages of Ni in them are only 30% to 40% or more, and a large part of the ingredients of the Ni-containing substances are CN_*x*_. If there exist central pipes filled with continuous Ni-containing substances and the resistivity of the Ni-containing substances is less than the CN_*x*_ bodies, the resistance of the CNNCs may be reduced; if not, the influence of the central pipes on the resistance of the CNNCs will be little. But, it is difficult to establish a model to estimate the extent of the influence due to their discontinuity and uncertain resistivity. Therefore, the resistivity of the CNNCs as a whole is calculated. As shown in Figure 4c,d, both the resistance and resistivity of the as-grown CNNCs are obviously affected by the CH_4_/N_2_ ratios. It could be found in Figure 4d that the resulted resistivity *ρ* decreases from 1.01 × 10^-3^ to 6.45 × 10^-5^ Ω · m as the CH_4_/N_2_ ratio increases from 1/80 to 1/5, which could be due to the increase of the carbon content in the CNNCs.

## Conclusions

In summary, the vertically aligned CNNC arrays were synthesized on nickel-covered silicon (100) substrates by the GPRD method. The morphologies and composition of the as-grown CNNC arrays are strongly affected by the CH_4_/N_2_ feeding gas ratios. The as-grown CNNCs are mainly amorphous CN_*x*_, and the atomic content of nitrogen decreases synchronously as the CH_4_/N_2_ ratio increases. The CNNC arrays grown at the CH_4_/N_2_ ratio of 1/5 have rather perfect cone shapes and good wettability to the polymer P3HT:PCBM. The absorption spectra reveal that the optical absorption of the as-grown CNNC arrays increases with increasing CH_4_/N_2_ ratio and show a very good absorption in a wideband of 200 to 900 nm at the CH_4_/N_2_ ratio of 1/5. The resistivities of the as-prepared samples decrease as the CH_4_/N_2_ ratios increase and reach about 6.45 × 10^-5^ Ω · m at the CH_4_/N_2_ ratio of 1/5, indicating that the as-grown CNNC arrays can have very good conductivity. Due to the large specific surface area, high and wide optical absorption, excellent electrical conduction, and nice wettability (to polymer absorbers) of the as-grown CNNC arrays, such nanocone arrays are supposed to be potential electrodes or even absorbers in the thin film solar cells and photodetectors.

## Abbreviations

CH4/N2: methane/nitrogen; CNNC: binary CN_*x*_ nanocone; EDXS: energy-dispersive spectroscopy; FESEM: field emission scanning electron microscopy; GPRD: abnormal glow discharge plasma-assisted reaction deposition; HRTEM: high-resolution transmission electron microscopy; PLD: pulsed laser deposition; P3HT:PCBM: poly-3-hexylthiophene mixed with phenyl-C61-butyric acid methyl ester; SAED: selected-area electron diffraction; TEM: transmission electron microscopy.

## Competing interests

The authors declare that they have no competing interests.

## Authors' contributions

XL designed and carried out the experiments and wrote the paper. LG, XF, and YZ participated in the experiments. JW participated in the design and the discussion of this study. NX conceived and designed the experiments and revised the paper. All authors read and approved the final manuscript.

## Authors' information

XL, LG, and XF are graduate students major in fabrication of nanometer materials. YZ is an associate professor and MS degree holder specializing in optical devices. JW is a professor and PhD degree holder specializing in optics and nanometer materials. NX is a professor and a PhD degree holder specializing in nanometer materials and devices, especially in nanoscaled super-hard and optoelectronic devices.
